# Headers and concussions in elite female and male football: a pilot study

**DOI:** 10.17159/2078-516X/2023/v35i1a15236

**Published:** 2023-03-06

**Authors:** S den Hollander, V Gouttebarge

**Affiliations:** 1 Football Players Worldwide (FIFPRO), Hoofddorp, The Netherlands; 2 Amsterdam UMC location University of Amsterdam, Department of Orthopedic Surgery and Sports Medicine, Meibergdreef 9, Amsterdam, The Netherlands; 3 Amsterdam Collaboration on Health & Safety in Sports (ACHSS), IOC Research Center, Amsterdam, The Netherlands; 4 Section Sports Medicine, University of Pretoria, Pretoria, South Africa; 5 Amsterdam Movement Sciences, Musculoskeletal Health, Sports, Amsterdam, The Netherlands

**Keywords:** soccer, professional, academy, ocular markers, force

## Abstract

**Background:**

Heading is a risk factor for neurogenerative disease in football. However, the exposure to heading in elite football training is understudied.

**Objectives:**

The primary purpose of this study was to determine the exposure to headers in elite men’s and women’s football and to describe the effects of the headers on ocular markers.

**Methods:**

Exposure to headers was observed over three days of women’s and men’s football. The number of headers at each session was determined through video analysis, and the G-force was determined via an impact tracker. Ocular markers were assessed at the start and end of the three days, and the results were compared to determine if there were any changes. Self-reported exposure to heading was recorded after each session and compared to the number of headers observed through video analysis, to assess the validity of players’ self-reporting.

**Results:**

Female players made an average of 11 headers per player per session. Ninety percent of the headers were below 10G, and none were above 80G. Male players made an average of three headers per player per session, with 74% of the headers recording a G-force above 10G and 3% above 80G. No meaningful changes were observed post-session in the ocular markers, and no concussions were observed. Neither cohort was able to accurately self-report exposure to headers.

**Conclusion:**

Longitudinal studies should be designed and conducted across different levels of play in both women and men’s football as a prerequisite to develop evidence-based measures to prevent or mitigate the potential risks associated with headers and concussions in elite football.

Research has indicated a potential causal link between repetitive head impacts and chronic traumatic encephalopathy, a neurodegenerative disease. ^[1]^ As such, concerns over the risk of cognitive decline and neurodegenerative diseases associated with heading in professional footballers has resulted in several attempts to limit the exposure to heading in training. ^[2, 3]^ However, in elite men’s and women’s football, not enough is known regarding the exposure to headers or their effect on cognitive function. This information is crucial to allow stakeholders to make informed decisions and intervene, where necessary, to improve players’ safety.

Research across five top men’s European leagues has found that defenders made approximately six headers per match, and midfielders and forwards four per match. ^[4]^ In elite women’s football, across positions, players make an average of four headers per player per match. ^[5, 6]^ In both women’s and men’s elite football, little is known about the exposure to headers in training. ^[7]^ This lack of data needs to be addressed, while the exposure to headers should be objectively quantified through, for example, video analysis, as subjective self-reported measures have been shown to be invalid. ^[8, 9]^

Along with quantifying the exposure to heading, it is also important to identify the force associated with heading in football. The force gives an indication of the impact a header had on a player’s brain. Studies in youth football, and in the laboratory, found the force of headers ranges between 4 and 50G, ^[7]^, well below the threshold of 80G associated with a concussive event. ^[10]^ It may, therefore, seem that the force associated with a header is not large enough to result in a concussion. However, little is known about the cumulative effect of heading on the brain. In other words, would the 6 headers of 50G a defender may make in a match (a cumulative force of 300G) affect their cognitive functioning, and could these sub-concussive events result in long-term decrements in brain health? The eye and brain share similarities in their neural and vascular structures and the immune response. ^[11]^ Assessing the health and functioning of the eye, and any changes in these assessments that may occur can provide insight into the health and functioning of the brain. ^[12, 13]^.

The primary objective of this study was twofold, namely, to (i) assess the exposure to headers and concussions among elite female and male footballers, and (ii) explore the effect of headers and concussions on their ocular markers. A secondary objective was to determine the validity of self-reported exposure to heading compared to video analysis.

## Methods

### Study design

An observational descriptive study design was conducted. Ethical approval was provided by the Medical Ethics Review Committee of the Amsterdam University Medical Centers (W22_016#22.045; Amsterdam, The Netherlands), while the study was conducted in accordance with the Declaration of Helsinki (2013).

### Setting

**Fig. 1:**
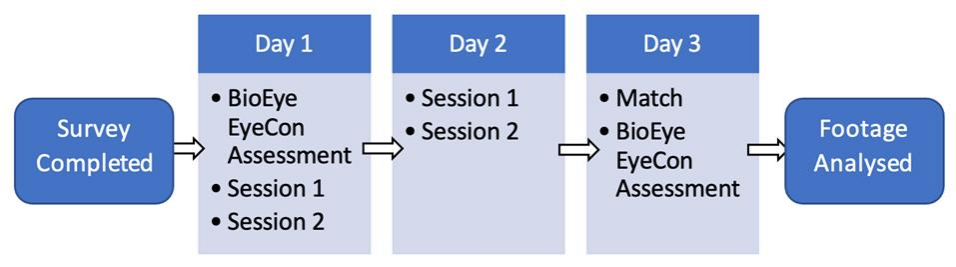
Flowchart of study protocol

This study was coordinated by FIFPRO (Football Players Worldwide), and a report of the study has been documented on their website (fifpro.org). ^[14]^ The women’s section of the study took place in June 2022 with the Women’s Israel Football Association’s academy (in collaboration with the Israeli Football Players Organisation IFPO; Israel) during their final training week of the season. The men’s section of this study took place in the 2^nd^ week of a training camp organised in March 2022 by Jalkapallon Pelaajayhdistys Ry, Football Association of Players in Finland (JPY). All sessions (Figure 1) were designed and executed by experienced coaches and no instruction was given to the coaches on the prescription of training.

### Participants

The participants consisted of two separate groups, namely, female and male elite footballers. In our study, the definition for an elite footballer was that he/she (1) trains to improve football performance; (2) competes in the highest or second highest national league; (3) reports football training and competition as their major activity, devoting several hours in all or most of the days for these activities, and (4) exceeding the time allocated to other types of leisure activities. Convenience sampling was used to recruit the participants; namely, the female group from players at the Women’s Israel Football Association’s academy and the male group from players at the JPY training camp. Based on a sample size calculation, 26 (13 per group) participants were required for the study. ^[15]^

### Measurements

#### Exposure to headers

Each session was filmed and all footage was analysed retrospectively by an experienced video analyst using a VLC Media Player (VideoLan Client). Each player was assigned a shirt with a unique identifiable number and an ACT Head Impact Tracker, which they wore during each session (act-tracker.com, Northern Sports Insight and Intelligence Oy). The tracker measured any impacts to the head over a force of 10G. Each observed header was noted and the timestamp of the header, whether the header was intentional, and the shirt number of the player was recorded. After each session, the players were also asked how many headers they thought they made during the session and their response was recorded. The timestamps of the impacts were aligned to the timestamps of the headers observed in the video footage, and the corresponding G-forces were recorded. Any impact forces not associated with a header were not included in the analyses.

#### Concussions

Any suspected concussions were identified by a side-line spotter and assessed by medical staff present at every session. If a suspected concussion was confirmed by the medical staff, video footage of the incident was reviewed to identify the mechanism and cause of the concussion.

#### Ocular markers

The ocular markers were assessed via the BioEye EyeCon device at the start and end of the training week (Bioeye.com). The measurements were conducted by a trained and experienced instructor. The EyeCon test battery consists of four components: smooth pursuit (SMP), pupillary light response (PLR), near point convergence (NPC), and horizontal gaze nystagmus (HGN). A description of each test, with normative values can be found in Appendix 1.

### Procedures

Potential participants were emailed the information about the objectives and procedures of the study by their respective national players union. If interested in the study, each participant gave their electronic consent and completed an electronic survey. The survey, compiled in English (Typeform Professional), consisted of several questions related to age, gender, height, weight, duration of their elite football career, studies and/or work outside of football, and field position. Once completed, the data was saved on a secured server that only the two principal investigators had access to. Each player was assigned a number, and no information regarding the identity of the player was recorded. Players executed all sessions and measurements as indicated previously. Players participated voluntarily in the study and did not receive any financial remuneration for their participation. Data were collected in February, March and June 2022.

### Statistical analysis

For our primary objective, descriptive statistics (average, maximum and minimum) were used to analyse the players' exposure to headers and concussive events, and the results of the Bio-Eye assessments. For our secondary objective, the interclass correlation coefficient (ICC) was used to determine the validity of self-reported exposure to headers compared to video analysis (Gold Standard). ICC values were evaluated as follows: ‘poor’ validity when ICC was lower than 0.50; ‘moderate’ validity when ICC ranged from 0.50 to 0.75, ‘good’ validity when ICC ranged between 0.75 and 0.90, and ‘excellent’ when ICC values were above 0.90. ^[16]^ IBM SPSS 26 and Microsoft Excel for Microsoft 365 were used to perform the data analyses.

## Results

### Participants’ characteristics

#### Women players

Sixteen elite academy-level women participated in the study (n=16; 2 goalkeepers, 5 defenders, 4 midfielders and 5 forwards). One hundred percent (100%) of the footballers were currently studying, and 6% were working outside of football. The age of the players ranged from 14 to 18 years, with an average and mode age of 16 years.

#### Men players

Fifteen professional male footballers participated in the study (n=15; 1 goalkeeper, 5 defenders, 7 midfielders and 3 forwards). Thirty-three percent (33%) of the footballers were currently studying, and 20% were working outside of football. The age of the players ranged from 22 to 33 years, with an average and mode age of 25 years and 22 years, respectively.

### Exposure to headers

#### Women players

**Fig. 2:**
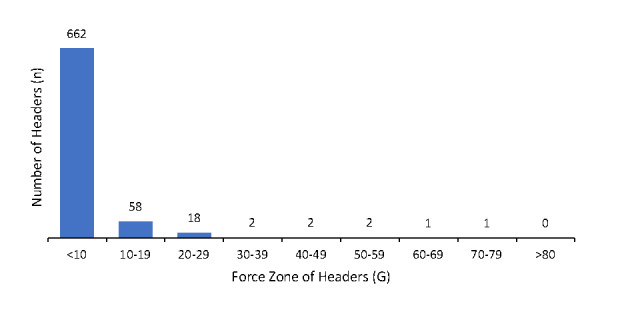
Number of headers made in each force zone in women’s football training

A total of 746 headers were observed over the five training sessions, with an average of 149 headers per session (11 headers per player per session). However, 87% (n=650) of the headers were made in Session 1 of Day 2. Due to player availability, the match on Day 3 was cancelled and replaced with an extra training session. Table 1 provides an overview of the average and maximum number and forces of the headers the players made. The force of the headers was not recorded during Day 1’s sessions (players needed to be familiarised with the system, and the system needed to be calibrated). Only 65 out of the 677 headers observed on Days 2 and 3 (10%) had a G-Force over 10G. The average force of the headers above 10G was 19G. No observed headers were above 80G in any of the sessions. There were two unintentional headers (0.3%), one had a G-Force of less than 10G and the second had a G-Force of 19G. Figure 2 provides a visual of the number of headers made in each force zone.

#### Men players

**Fig. 3:**
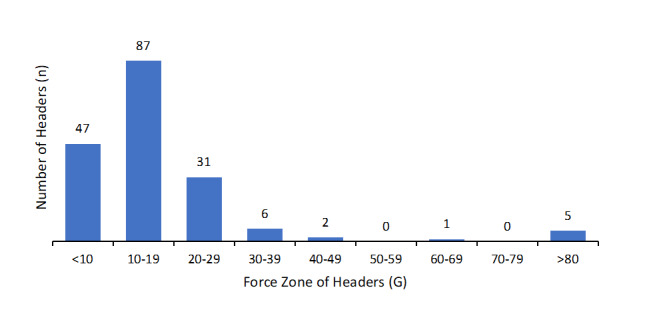
Number of headers made in each force zone in men’s football training and match

There was a total of 179 headers observed over the five sessions, with an average of 35 headers per training session (two headers per player per session) and 38 headers in the match (three headers per player). One hundred and thirty-two (132) out of the 179 headers had a force over 10G, 72% in training and 82% in matches. The average force of the headers above 10G was 19G in training, and 29G in matches. There were 175 intentional headers, with an average force of 19G, and four unintentional headers, with an average force of 77G. Three headers in training, and two in the match had a G-force above 80G. An overview of the average and maximum number and forces of headers, and a visual of the number of headers made in each zone are shown in Table 2 and Figure 3, respectively.

### Concussions, ocular markers and validity of self-report

There were no observed concussions at any of the women’s or men’s training or match sessions.

An overview of the results of the EyeCon assessments, for women and men, are presented in Table 3, with average scores and standard deviation. For both groups, the results of the changes in the SMP, PLR, and NPC tests were within the normative range, suggesting no clinically relevant acute changes in cognitive functioning. There were no cases of HGN detected in either the women’s or men’s group.

The validity of the women players to self-report the number of headers they made during training ranged from *poor* to *excellent* between the sessions (Table 1), and from *poor* to *good* for the men players (Table 2).

## Discussion

The purposes of this study were (i) to assess the exposure to headers and concussions among elite footballers and (ii) to explore the effect of headers and concussions on their ocular-markers. A secondary objective was to determine the validity of self-reported exposure to heading, compared to video analysis. Female players made an average of 11 headers per player per session. Ninety percent of the headers were below 10G, and none were above 80G. Male players made an average of three headers per player per session, with 74% of the headers recording a G-force above 10G and 3% above 80G. There were no clinically relevant changes observed post-session in the ocular markers of the players, and no concussions were observed. Neither the women’s nor men’s football cohort were able to accurately self-report the number of headers they made in every session.

### Exposure to headers

There was an average of 11 headers per player per training session in women’s football, and an average of two and three headers per player per session in training and matches in men’s football, respectively. The exposure to headers in men’s football is similar to that reported in previous studies in men’s and women’s collegiate and elite football, with an average of two headers per player per training session, ^[6, 17, 18]^ and four headers in matches, ^[5, 18]^ reported in both men’s and women’s football. Although the number of headers we identified in women’s football was higher, 87% of these headers occurred in one session. If we exclude that outlying session from the calculation, there was an average of two headers per player per session in women’s football. This highlights the impact the design of a training session can have on the players’ exposure to heading, and a possible need to educate coaches on the importance of designing training sessions that limit the exposure of heading in training, to reduce the potential risk of repetitive headers on brain health.

The average force of headers (calculated with those above 10G) in men and women’s football training was 19G. Similar findings were found in collegiate players in a study by Saunders and colleagues, with an average force of 20G and 17G for women and men players, respectively. ^[19]^ However, the percentage of headers above 10G, and the number of headers above 80G, was higher in men’s football when compared to women’s football. Head impacts above 80G have been identified as a risk factor for concussion. ^[20]^ Although our findings suggest that the men were exposed to headers with greater impacts than women, it is important to note that the men’s group was older than the women’s (senior vs academy). Therefore, further research is required to describe the exposure to heading in men’s and women’s football, at different levels of play and different career stages. Although 3% of headers in the men’s group were above 80G, the impacts did not cause a concussion. The risk of concussion may have been mitigated by a player’s well-developed neck musculature or proficient heading technique ^[21]^; however, this investigation was beyond the scope of this study and research investigating the mechanism of heading-related concussions is recommended.

### Ocular markers

The exposure to headings over the three days of training, in women’s and men’s football, did not have a clinically relevant effect on the players’ ocular markers. A review of research related to heading in football found that in 75% of the studies reviewed, heading did not have a negative effect on cognitive functioning. ^[5]^ These findings suggest that the impact of headings may not have an acute effect on cognitive functioning. However, longitudinal studies are required to determine the chronic effect the impact of headings may have on cognitive functioning across multiple months or years of training and matches.

### The validity of self-report

The validity of the players ability to self-report how many headers they made in a session ranged from poor to excellent, and poor to good, in women’s and men’s football, respectively. It is, therefore, recommended that in future research objective measures (i.e. video analysis) are used to quantify players exposure to heading in training and matches.

### Limitations

The purpose of a pilot study is to examine the methodology and procedures of a study on a small scale, and to explore the feasibility of the study and identify any limitations and considerations, before implementing the study on a larger scale. In this sense, this study was a success as several limitations and considerations were identified which can inform future directions.

We observed the exposure to headings in men and women’s football. Yet, although both groups compete at an elite level, the level of play, career stage, and the country where they played also differed between the groups. These factors may affect the level and style of training of the players, and the subsequent heading exposure. This can make it difficult to attribute differences between groups to a specific grouping factor (sex, level of play, etc.). We therefore recommend that future studies which analyse both men and women’s football consider the level of play, the country, and the career stage of the players.

There were a few technical errors related to the synching of the timestamps of the head impacts with the video footage. Namely, the time stamps from the head impacts during the men’s training camp were incorrect due to a software issue. This issue was fixed for the women’s training, but the video footage of the training was not time stamped. This meant that, although we were able to quantify the number of headers (and any other head impacts) each player made in a session, and the force of the head impacts that occurred, we were not able to objectively know the force of each header. Future studies should therefore ensure that the impact trackers used are able to accurately record impacts from heading and that the recordings of training and matches is timestamped.

Convenience sampling was used for our recruitment of participants. Therefore sampling bias must also be considered a limitation of the study.

### Future directions

The Sequence of Injury Prevention is a sports injury prevention model that was developed to provide researchers and practitioners with a road map to guide decision-making and action plans when developing injury prevention strategies. ^[22]^ The first two steps of this model are (1) to establish the extent of the problem, (2) to establish the mechanism of injury, and to introduce preventative measures. We recommend that future studies start with the first two steps of this model, namely, to establish players' exposure (number and force) to head impacts in training and matches, and the incidence, severity, and mechanisms of changes in cognitive function (e.g. via ocular markers) in elite football. Therefore, observational longitudinal (over at least one season) studies should be designed and conducted across different levels of play, in both women's and men’s football. These studies are a prerequisite before the development of any evidence-based measures aiming to prevent or mitigate the potential risks associated with headers and concussions in elite football.

## Conclusion

Female players made an average of 11 headers per player per session. Ninety percent of the headers were below 10G, and none were above 80G. Male players made an average of 3 headers per player per session, with 74% of the headers recording a G-force above 10G and 3% above 80G. There were no significant changes observed post-session in the ocular markers of the players, and no concussions were observed. Neither the women’s nor men’s football cohorts were able to accurately self-report the number of headers they made in every session.

## Supplementary Information



## Figures and Tables

**Table 1:** Number and force of headers in women’s football training (n=16)

	Day 1		Day 2		Day 3
**Session**	1	2	1	2	1
**Maximum headers observed by a single player (n)**	7	2	72	10	3
**Average headers observed per player (n)**	4	<1	46	2	1
**Average self-reported headers per player (n)**	11	1	33	2	1
**Intraclass correlation (ICC)**	0.11	0.73	0.49	0.94	0.88
**ICC interpretation**	Poor	Good	Moderate	Excellent	Good
**Average number of headersabove 10G per player (n)**			4	2	<1
**Average g force of headersabove 10G (G)**			19	17	17
**Number of headers above 80G (n)**			0	0	0
**Maximum g force of a header (G)**			71	27	22

**Table 2:** Number and force of headers in men’s football training and match (n=15)

	Day 1		Day 2		Match
Session	1	2	1	2	1
**Maximum headers observed by a single player (n)**	3	16	15	3	5
**Average headers observed per player (n)**	<1	8	1	1	3
**Average self-reported headers per player (n)**	1	6	3	2	3
**Intraclass correlation (ICC)**	0.07	0.77	−0.14	0.48	0.28
**ICC interpretation**	*Poor*	*Good*	*Poor*	*Poor*	*Poor*
**Average number of headers above 10G per player (n)**	<1	6	1	<1	2
**Average g force of headers above 10G (G)**	11	17	34	32	27
**Number of headers above 80G (n)**	0	1	1	1	2
**Maximum g force of a header (G)**	13	101	111	94	137

**Table 3:** Changes in Bioeye Eyecon Ocular Markers between Day 3 and Baseline assessment results

Ocular assessments	Baseline				Day 3				Change			

	Women		Men		Women		Men		Women		Men	
	Mean	SD	Mean	SD	Mean	SD	Mean	SD	Mean	SD	Mean	SD

**Left eye SMP latency (ms)**	321	89	317	48	304	39	293	52	−17	108	−24	−37
**Right eye SMP latency (ms)**	279	66	297	63	291	46	269	56	12	63	−28	76
**Left pupil size (mm)**	5.9	1.2	5.3	0.5	5.6	0.8	5.4	0.5	−0.3	1.5	0.1	0.6
**Right pupil size (mm)**	5.9	1.1	5.4	0.6	5.5	0.8	5.4	0.5	−0.4	1.1	0	0.5
**Difference in pupil size (mm)**	0.1	0.4	0.1	0.3	0.1	0.3	0.1	0.3	0.0	0.5	0	0.4
**Total pupil constriction (mm)**	1.7	0.7	1.5	0.6	1.4	0.5	1.5	0.4	−0.3	1.0	0	0.4
**NPC loss of convergence (cm)**	9.0	3.0	9.6	3.2	9.1	3.6	9.0	2.7	0.1	5.7	−0.6	4.2
**NPC regained convergence (cm)**	9.0	2.9	9.0	2.7	9.0	2.7	9.1	2.1	0	4.7	0.1	3.0

SMP, smooth pursuit; NPC, near point convergence
