# Supplementary material for: Headers and concussions in elite female and male football: a pilot study
Source: S Afr J Sports Med. 2023 Mar 6;35(1):v35i1a15236. doi: 10.17159/2078-516X/2023/v35i1a15236 (PMC10798605; doi:10.17159/2078-516X/2023/v35i1a15236)
Supplement: Supplementary file 1 [file 2078-516X-35-v35i1a15236-s001.pdf]

Headers and concussions in elite female and male football: a pilot study

Appendix 1: EyeCon Test Battery (Bioeye.com)

Smooth Pursuit

The SMP assesses how the eyes respond to the movement of a tracking target. In comparison to healthy individuals, concussed individuals tend to have greater latency in responding to the movement of a tracking target. The norm values for this test are between 200 and 250ms, and an increase of 50ms from the baseline measurement may imply a concussive state.

Pupillary light response

The PLR test measures the response of the eye to a light stimulus. The size of each pupil was measured before the stimulus when dilated, and after the stimulus, when totally constricted. The norm size of a dilated pupil is between 4 and 6mm, and an increase of 1.5mm from the baseline measurement may imply a concussive state. An asymmetry in dilated pupil size (difference between left and right) of more than 0.5mm from the baseline measurement may also imply a concussive state. The norm size of a constricted pupil is between 1 and 2mm, and an increase of 1mm from the baseline measurement may imply a concussive state. The PLR test was repeated at the end of the EyeCon test battery.

Near Point Convergence

For the NPC test an object is moved towards and away from your face until your eyes lost and regained convergence. The norm values for the loss of convergence are 7-9cm and regained convergence is 10-15cm, and an increase of 3mm from the baseline measurement for both lost and regained convergence may imply a concussive state.

Horizontal Gaze Nystagmus

The HGN test screens for nystagmus, a condition where the eyes make repetitive, involuntary movements, which result in poor vision and depth perception.
